# Mathematical Formulation of DMH-Based Inverse Optimization

**DOI:** 10.3389/fonc.2014.00331

**Published:** 2014-11-17

**Authors:** Ivaylo B. Mihaylov, Eduardo G. Moros

**Affiliations:** ^1^Department of Radiation Oncology, University of Miami, Miami, FL, USA; ^2^Department of Radiation Oncology, H. Lee Moffitt Cancer Center, Tampa, FL, USA

**Keywords:** dose, volume, mass, inverse, optimization

## Abstract

**Purpose:** To introduce the concept of dose–mass-based inverse optimization for radiotherapy applications.

**Materials and Methods:** Mathematical derivation of the dose–mass-based formalism is presented. This mathematical representation is compared to the most commonly used dose–volume-based formulation used in inverse optimization. A simple example on digitally created phantom is presented. The phantom consists of three regions: a target surrounded by high- and low-density regions. The target is irradiated with two beams through those regions and inverse optimization with dose–volume and dose–mass-based objective functions is performed. The basic properties of the two optimization types are demonstrated on the phantom.

**Results:** It is demonstrated that dose–volume optimization is a special case of dose–mass optimization. In a homogenous media, dose–mass optimization turns into dose–volume optimization. The dose calculations performed on the digital phantom show that in this very simple case dose–mass optimization tends to penalize more the dose delivery through the high-density region and therefore it results in delivering more dose through the low-density region.

**Conclusion:** It was demonstrated that dose–mass-based optimization is mathematically more general than dose–volume-based optimization. In the case of constant density media, dose–mass optimization transforms into dose–volume optimization.

## Introduction

Modern radiotherapy treatment planning relies on the dose–volume-histogram (DVH) paradigm, where doses to volumes of anatomical structures are employed (1–4). The widespread use of DVHs is rooted within the wealth of clinical information as well as clinician’s experience with dose–volume metrics (5). DVHs were introduced more than three decades ago, while intensity modulated radiotherapy (IMRT) was developed a decade later (4, 6–10). At those times, homogeneous dose calculations were the norm, with heterogeneous dose calculations hardly even possible, and therefore, not practical for a routine use. In recent years, however, it has been argued that the effects of delivered dose seem to be more closely related to healthy tissue toxicity (and thereby to clinical outcomes) when dose to mass, or dose–mass-histograms (DMHs), are considered in treatment plan review and evaluation (11–16).

Dose–mass-histograms were introduced for evaluation and review of thoracic treatment plans (15, 16). Shortly, after their introduction, a rationale for their application was outlined (11, 12). In those publications, it was argued that DVHs of the lungs are breathing phase dependent, while DMHs are not (11). Investigation on the difference between DVHs and DMHs and their effects on the treatment outcomes showed that the range of deviation between them is very large (12). It was concluded that “*the effectiveness of the dose distribution delivered to the patients seems to be more closely related to the radiation effects when using the DMH concept*” (12). Similar conclusions that “*DMH may be more relevant than DVH*” were drawn in an investigation of DVH and DMH effects on 4D lung treatment plans (14).

## Mathematical Framework of DMH Inverse IMRT Optimization

Consider DVH-based IMRT optimization, where plans are designed through a number of dose–volume objectives (4, 17, 18). The optimization algorithm divides each beam’s cross-sectional plane into a 2D-array of finite size beamlets, which initially are assigned equal weights. With the initiation of the optimization those weights are varied (optimized), such that 2D intensity maps of variable intensities are created, with the aim of maximizing dose to targets, while at the same time minimizing doses to adjacent organs at risk (OARs). The doses to all volumes of interest (VOIs), resulting from those intensity maps from all beams, give rise to a set of optimization functions *F^j^, j* = 1, … , *n*, where *j* runs over all the objectives specified for all VOIs, including targets and OARs. Those optimization functions are a mathematical representation of

(1) $$F=\sum_{j=1}^{N}F^{j}$$

IMRT objectives. The inverse optimization algorithm aims in minimizing a composite objective function given by Eq. 1. This function is a sum of all individual optimization objectives.

(2) $$F^{j}=\sum_{i\in V}{\left(\frac{d_{i}-d^{j}}{d^{j}}\right)}^{2}\Delta v_{i}$$

For each VOI there might be, none or more than one *F^j^* specified, depending on the aims of the radiotherapy plan. Equation 2 describes an example of a quadratic objective function (18). *V* denotes the volume of the VOI for which *F^j^* is evaluated, *d_i_* is the dose in voxel (3D volume element) *i, d^j^* is the desired dose in each voxel, and *v_i_* is the normalized (with respect to the entire VOI volume) voxel volume. The summation can be over the entire (min/max dose objectives) or partial (DVH-objectives) volume *V* of the VOI. The quadratic term in Eq. 2 makes the functions *F^j^* always positive, thereby requiring the optimization to find only a minimum, i.e., to minimize the differences between individual voxel doses and the desired dose for the specified objective. The normalization with respect to the desired dose *d^j^* and to total organ volume in the equation terms, respectively, scales all functions *F^j^* such that the contributions from targets and OARs in the global optimization of *F* are of the same magnitude and a global composite objective function (cf. Eq. 1) can be constructed. Note that, if the voxels of the dose grid (cf. Eq. 2) are of equal volumes the corresponding Δ*v_i_* for most of the voxels will be the same, and can be moved in front of the summation. This makes the sum of Eq. 2 only partially dependent on volumes Δ*v_i_*. This partial dependence is because of partial volume effects, where given VOI occupies only a fraction of a given dose voxel *i*, and Δ*v_i_* for that dose voxel is different from Δ*v_i_* for the dose voxels, which are fully contained in the VOI.

The implementation of tissue mass information and converting volume-based optimization into mass-based optimization can be achieved through Eq. 3, where the last term represents the voxel mass, normalized to the total VOI mass.

(3) $$F^{j}=\sum_{i\in V}{\left(\frac{d_{i}-d^{j}}{d^{j}}\right)}^{2}\Delta m_{i}$$

If the mass term in Eq. 3 is expanded then the mass-based objective function will be represented by Eq. 4, where *ρ_i_* is the averaged density in voxel *i*. Usually, the dose voxels in

(4) $$\begin{array}{llll} F^{j}=\sum_{i\in V}{\left(\frac{d_{i}-d^{j}}{d^{j}}\right)}^{2}\Delta m_{i} & =\sum_{i\in V}{\left(\frac{d_{i}-d^{j}}{d^{j}}\right)}^{2}\; & & \;\\ & \;\times \frac{\rho_{i}\times v_{i}}{\sum_{k\in V}\rho_{k}\times v_{k}} \end{array}$$

radiotherapy treatment planning are much larger than the voxels of the of the underlying computed tomography (CT) data. Therefore, the density in the dose voxel is an averaged from the CT density derived from the raw CT data through a CT-to-density calibration tables.

It follows from Eq. 4 that in the situation where the density in all dose voxels is constant (i.e., CT scan of a uniform density object), there should be no difference between DVH-based and DMH-based optimizations, since Eq. 4 would be transformed into Eq. 2. Constant density *ρ* = *ρ_i_* _=_ *ρ_k_* can be moved in front of the summation in Eq. 4 and it will cancel out. Therefore, mass-based optimization for heterogeneous media will naturally remove a degree of degeneracy, inherent to volume-based optimization. It must be stressed out that from mathematical and physical stand points DMH-based optimization is a more general approach than DVH-based optimization in radiotherapy applications. If the density across a VOI is variable, Δ*m_i_* in Eq. 4 will change from voxel-to-voxel in addition to partial volume effects mentioned above. This difference in the functional forms of the optimization functions *F^j^* will result in IMRT solutions for DMH optimization, which may differ from the solutions achieved through DVH optimization.

## Example

**Figure 1:**
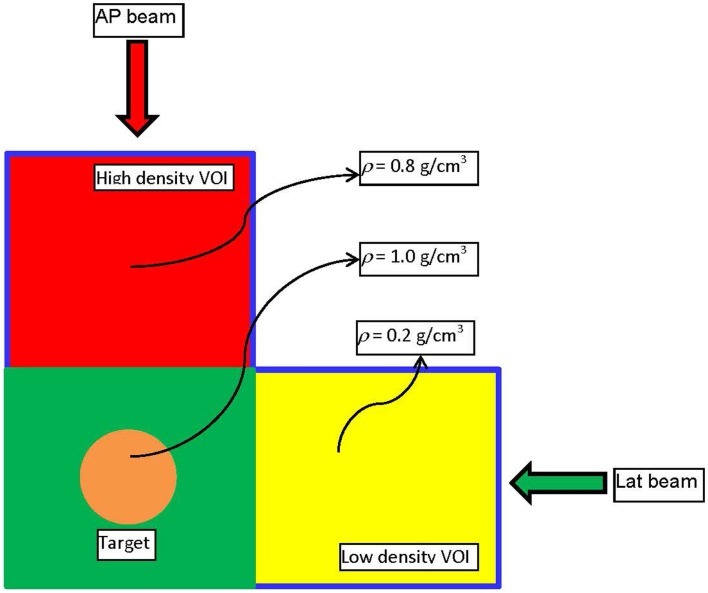
**The experimental set-up used to demonstrate the dose–mass concept**. It is realized through a digitally constructed phantom, consisting of three cubical volumes (VOIs) with dimensions 10 cm × 10 cm × 10 cm. As depicted on the figure, the three regions have different densities. In the middle of the VOI with density of unity, there is a cylindrical target with height and diameter of 3 cm.

A simple example will be presented to illustrate the basic points of the derived framework for mass-based optimization and to outline the differences with dose–volume-based optimization. Consider the experimental set-up presented on Figure 1. The figure depicts a digital phantom in an axial view. The phantom consists of three 10 cm × 10 cm × 10 cm cubes with densities of 0.2 (yellow), 0.8 (red), and 1.0 (green) g/cm^3^, respectively. In the middle of the green VOI, there is a cylindrical target with diameter and length of 3 cm. The target was irradiated with an anterior–posterior (AP) and a lateral (Lat) beam centered on the geometric center (isocenter) of the target. In the first experiment, target was irradiated with the AP and the Lat beams through 2 cm × 2 cm open apertures with the goal to deliver 500 cGy to 95% of the volume. The weights of the two beams were set equal. 833 monitor units (MUs) were required for that target dose prescription to be achieved. 474 MUs were delivered through the high-density (red) region, while, not surprisingly, only 359 MUs were delivered through the low density (yellow). In other words, 57% of the dose came through the high-density region and 43% was delivered through the low-density region, since the absorption in the high-density region is larger.

**Figure 2:**
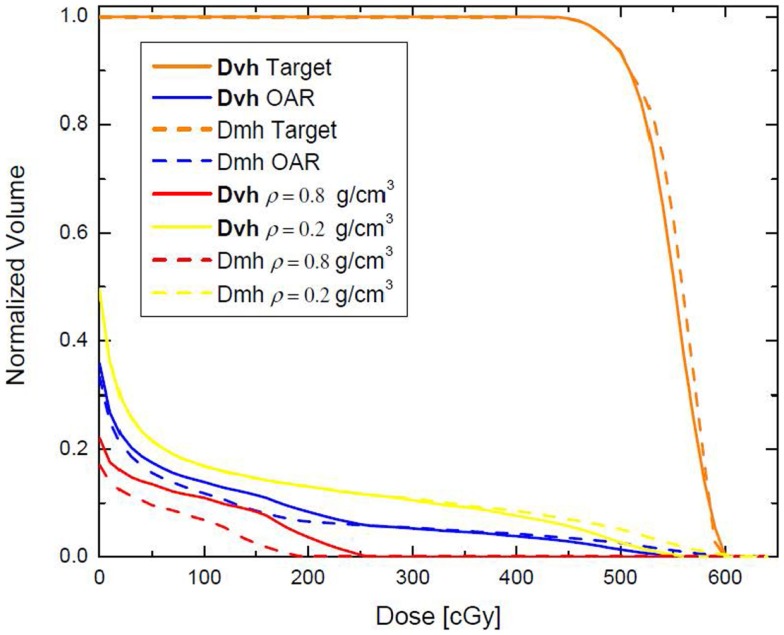
**Dose–volume histograms, resulting from DVH and DMH optimization applied to the phantom presented on Figure 1**. The high- and low-density VOIs have been combined in a single VOI with the aim that 500 cGy are delivered to the target, while the dose to that VOI is minimized as much as possible.

The same phantom is used in a different example where the high (red) and low (yellow) density regions are combined to form an “organ at risk” (OAR) to which the dose should be minimized through an inverse optimization. The two beams – AP and Lat – were allowed to have only one IMRT segment each. Two plans were generated – one where the cost function for OAR dose optimization was constructed according to Eq. 2, and another one where the OAR dose optimization was based on Eq. 3. Those optimizations were termed DVH and DMH, respectively. With each optimization the dose to the OAR was iteratively decreased until the standard deviation of the dose across the target reached 6% of the prescription dose, i.e., no more than 30 cGy. The dose–volume histograms (DVHs) of the two optimization approaches are presented on Figure 2. It is evident form the figure that while the high-tail dose to the low-density region (yellow) is higher with DMH optimization, the overall DVH for the higher density region is lower than in the case of DVH optimization. In the DVH optimization, 26.86% of the MUs were delivered through the higher density region, while the rest 73.14% were delivered through the lower density region. For the DMH optimization those percentages were 20.62 and 79.38, respectively. Therefore, optimization based on masses of the VOIs will penalize more the beams contributing dose through the high-density region (AP beam) rather than through the low density (Lat beam), given that the objective for the optimization is to minimize the dose delivered to both high- and low-density VOIs simultaneously. Effectively, for certain target coverage, more radiation would be delivered through the low-density region and less through the high-density VOI.

## Conclusion

A new framework for dose–mass optimization paradigm in inverse radiotherapy treatment planning was presented. It was shown through a mathematical derivation that dose–volume-based optimization is a special case of its more general representation realized through dose–mass optimization. In other words, dose–mass optimization transforms in dose–volume optimization in the case of constant density media. Simple computational example was presented to explain the basic properties of the two optimization types.

## Conflict of Interest Statement

The authors declare that the research was conducted in the absence of any commercial or financial relationships that could be construed as a potential conflict of interest.
